# Two-Dimensional Image Lempel–Ziv Complexity Calculation Method and Its Application in Defect Detection

**DOI:** 10.3390/e27101014

**Published:** 2025-09-27

**Authors:** Jiancheng Yin, Wentao Sui, Xuye Zhuang, Yunlong Sheng, Yongbo Li

**Affiliations:** 1 School of Mechanical Engineering, Shandong University of Technology, Zibo 255049, China; suiwt@163.com (W.S.); zxye@sdut.edu.cn (X.Z.); shengyunlong@sdut.edu.cn (Y.S.); 2 School of Aeronautics, Northwestern Polytechnical University, Xi’an 710072, China; 3 Modern Agricultural Equipment Research Institute, Shandong University of Technology, Zibo 255049, China

**Keywords:** Lempel–Ziv complexity, two-dimensional image, defect detection, local receptive field

## Abstract

Although Lempel–Ziv complexity (LZC) can reflect changes in object characteristics by measuring changes in independent patterns in the signal, it can only be applied to one-dimensional time series and cannot be directly applied to two-dimensional images. To address this issue, this paper proposed a two-dimensional Lempel–Ziv complexity by combining the concept of local receptive field in convolutional neural networks. This extends the application scenario of LZC from one-dimensional time series to two-dimensional images, further broadening the scope of application of LZC. First, the pixels and size of the image were normalized. Then, the image was encoded according to the sorting of normalized values within the 4 × 4 region. Next, the encoding result of the image was rearranged into a vector by row. Finally, the Lempel–Ziv complexity of the image could be obtained based on the rearranged vector. The proposed method was further used for defect detection in conjunction with the dilation operator and Sobel operator, and validated by two practical cases. The results showed that the proposed method can effectively identify independent pattern changes in images and can be used for defect detection. The accuracy rate of defect detection can reach 100%.

## 1. Introduction

The complexity of the time series reflects the nonlinear dynamic characteristics contained in the sequence and further reflects the dynamic behavior or health status of objects [1]. Thus, the capture and analysis of object state changes can be implemented by analyzing the complexity changes in time series.

According to Kolmogorov complexity theory, the complexity of a time series can be replaced by the complexity of a symbol sequence and the complexity of the symbol sequence can be defined as the shortest length that can reproduce the sequence [2,3,4]. On this basis, Lempel and Ziv [5] defined the complexity of random sequences in their research on information theory in 1976, stating that complexity reflected the rate at which new patterns appeared in a given sequence as its length increased, and reflected the degree to which the sequence approached randomness. The complexity algorithm for sequences of elements belonging to a finite set was proposed [5], which mathematically turned the calculation of Kolmogorov complexity into reality. The core idea of the Lempel–Ziv complexity algorithm was to reflect the complexity of the time series by analyzing repeated patterns in the time series. In a time series, the repeating pattern referred to the same subsequence that had appeared in different positions. For example, in the string “ABABAB”, “AB” was a repeating pattern because it appeared repeatedly in different positions. Since its introduction, Lempel–Ziv complexity has been widely applied in various fields [6,7,8,9], which can effectively reflect the independent pattern change in time series (abnormal data and points were equivalent to new independent patterns appearing). Currently, Lempel–Ziv complexity is mainly used for medical signal analysis [10,11,12] and mechanical equipment signal analysis [1,13,14]. The traditional process of calculating Lempel–Ziv complexity compared the original time series with a predefined threshold (usually the median or mean of the time series), which was labeled as 1 when the threshold was exceeded, and 0 otherwise. The original time series was converted to a 0–1 symbol sequence. However, this process resulted in the loss of a lot of information from the original time series, which could easily cause “over coarse-graining”, confuse the randomness and chaotic characteristics of the time series, and even change the dynamics of the original time series, resulting in the complexity analysis producing inaccurate results.

To make the encoded symbol sequence reflect more effective information in the original time series, Zhang et al. [15,16] proposed a 3-bit encoding method to encode the original time series and use it for EEG signal detection. Yin et al. [17] used symbolic aggregate approximation (SAX) to reflect the modulation information of bearing signals and encoded bearing signals to achieve fault severity recognition of bearing. In addition to the above improvement methods for Lempel–Ziv complexity encoding, the theory of entropy was used to achieve improvements in Lempel–Ziv complexity encoding. Many scholars have used the theories of permutation entropy and dispersion entropy to encode the original time series and proposed permutation Lempel–Ziv complexity (PLZ) and dispersion Lempel–Ziv complexity (DLZ). For example, Bai et al. [18] combined permutation entropy and Lempel–Ziv complexity for quantifying the dynamical changes in EEG. Bai et al. [19] used PLZ to describe the changes in EEG and quantify the effects of GABAergic anesthetics on brain activity. Shumbayawonda et al. [20] used PLZ to estimate the complexity of magnetoencephalography signals to research the changes in resting state brain (magnetic) activity caused by healthy aging. Li et al. [21] proposed DLZ for bearing fault diagnosis and live types of ship radiation noise signal classification. Jiao et al. [22] introduced DLZ into the field of underwater acoustics and fault diagnosis and proposed a feature extraction method for ship and gear fault signals based on DLZ. Although the above methods improved the accuracy of Lempel–Ziv complexity in characterizing object characteristics by changing the Lempel–Ziv encoding method, they only focused on the information of objects at one scale and cannot reflect the state of objects more comprehensively.

Therefore, the multiscale analysis method of time series was used to preprocess the original time series to further improve the accuracy of Lempel–Ziv complexity in reflecting object characteristics. The most commonly used multiscale analysis methods were coarse graining operation [23] and hierarchical analysis [24], and multiscale symbolic Lempel–Ziv complexity [25], multiscale permutation Lempel–Ziv complexity [26,27], multiscale dispersion Lempel–Ziv complexity [28,29,30], hierarchical Lempel–Ziv complexity [31], and hierarchical dispersion Lempel–Ziv complexity [14] were proposed by combining the Lempel–Ziv complexity under different encodings. In order to eliminate the problem of increasing scale and decreasing sequence length, refined composite multiscale Lempel–Ziv complexity [32,33,34] was further proposed. In addition, other multiscale methods have also been used in combination with Lempel–Ziv complexity. For example, Li et al. [35] proposed an unequal-step multiscale analysis to solve the problem of decreasing subsequences with increasing scale in existing coarse-graining methods and further proposed unequal-step multiscale integrated mapping dispersion Lempel–Ziv complexity. Li et al. [36] proposed refined time-shift multiscale dispersion Lempel–Ziv complexity by constructing the time-shift multiscale sequence. Utilizing multiscale Lempel–Ziv complexity can more comprehensively reflect the characteristics of objects.

In addition, many denoising methods have been used to preprocess the original time series to eliminate the impact of noise on Lempel–Ziv complexity in response to the problem of noise in mechanical equipment signals. These denoising methods can be further divided into frequency lossy noise reduction methods [37,38,39,40,41] and frequency lossless noise reduction methods [42]. For example, Noman et al. [43] improved the accuracy of Lempel–Ziv complexity in determining the health status of bearing by reducing the noise of the original signal using a continuously adjustable parameterized tunable Q factor wavelet transform. Wang et al. [44] improve the bearing fault severity recognition accuracy of Lempel–Ziv complexity by utilizing optimized variational mode decomposition. Yin et al. [13] utilized manifold learning to reduce noise in the signal and improved the recognition accuracy of bearing fault severity. Reducing the noise of the original signal can effectively reduce the effect of noise on the Lempel–Ziv complexity and improve the accuracy of the Lempel–Ziv complexity in reflecting the bearing condition.

Although changing the encoding method, increasing the scale of analysis, and reducing noise in the signal can effectively improve the accuracy and comprehensiveness of Lempel–Ziv complexity in reflecting object states, all the above methods can only calculate the Lempel–Ziv complexity of one-dimensional time series and cannot calculate the Lempel–Ziv complexity of two-dimensional image signals. However, in the process of detecting defects in images, it was also necessary to determine whether there were new patterns appearing in the image.

To address the above issue, this paper proposed a two-dimensional Lempel–Ziv complexity by combining the concept of local receptive field in convolutional neural networks. First, the pixels and size of the image were normalized to eliminate the impact of image size on Lempel–Ziv complexity. Then, the image was encoded according to the sorting of normalized values within the 4 × 4 region. Next, the encoding result of the image was rearranged into a vector by row. Finally, the Lempel–Ziv complexity of image can be obtained based on the rearranged vector. The main contribution of this paper was to propose a method for calculating the Lempel–Ziv complexity of two-dimensional images, further expanding the application scope of Lempel–Ziv complexity.

The remaining sections of the paper are organized as follows: Section 2 provides the necessary background knowledge of Lempel–Ziv complexity. Two-dimensional Lempel–Ziv complexity is proposed in Section 3. In Section 4, one-dimensional and two-dimensional simulation signals are used to demonstrate the ability of the proposed method to reflect the component changes in the signals. The morphological dilation operator and Sobel operator are combined with the proposed method for defect detection and verified with two practical cases, and the limitations of the proposed method are further discussed. The conclusions of the paper are presented in Section 5.

## 2. Background Knowledge of Lempel–Ziv Complexity

Lempel–Ziv complexity is widely used in the medical [11,45,46] and industrial [8,47] fields to measure the complexity of one-dimensional time series. It extracts the independent patterns from one-dimensional time series by copying and inserting operations [37], and determines the complexity of the time series based on the number of independent patterns. For a time series $X=\left\{x_{1},x_{2},\ldots x_{N}\right\}$, the Lempel–Ziv complexity can be calculated as follows:

Step 1: Calculate the mean of the time series as follows:

(1) $$\mu_{O}=\frac{1}{N}\sum_{i=1}^{N}x_{i}$$

Step 2: Encode the time series based on the relationship between time series and the mean $\mu_{O}$.

(2) $$s_{i}=\left\{\begin{array}{l} 1\;\;x_{i}\geq \mu_{O}\\ 0\;\;x_{i}<\mu_{O} \end{array}\right.$$

Step 3: Initialize parameters $S_{v,0}=\{\;\}$, $Q_{0}=\{\;\}$, $C_{N}=0$, r=1.

Step 4: Take $Q_{r}=\left(Q_{r-1}s_{r}\right)$ and judge if $Q_{r}$ belongs to $S_{v,r-1}=\left\{S_{v,r-2}s_{r-1}\right\}$. If $Q_{r}\notin S_{v,r-1}$, then set $Q_{r}=\left\{\;\right\}$, $C_{N}\left(r\right)=C_{N}\left(r-1\right)+1$, r=r+1. If $Q_{r}\in S_{v,r-1}$, then set $Q_{r}=\left(Q_{r-1}s_{r}\right)$, $C_{N}\left(r\right)=C_{N}\left(r-1\right)$, r=r+1.

Step 5: Repeat Step 4 until the time series is completely covered. And the $C_{N}\left(N\right)$ is the number of independent patterns in the time series.

Step 6: Normalize $C_{N}\left(N\right)$ by the following equation to eliminate the impact of time series length on $C_{N}\left(N\right)$.

(3) $$0\leq C_{nN}=\frac{C_{N}\left(N\right)}{C_{UL}}\leq 1$$

where

(4) $$C_{UL}=\underset{N\to \infty }{\lim}C_{N}\left(N\right)=\underset{N\to \infty }{\lim}\frac{N}{\left(1-\beta \right)\log_{k}N}\approx \frac{N}{\log_{k}N}$$

where k is the size of the alphabets used in the string $S_{N}=\left\{s_{1}s_{2}\ldots s_{N}\right\}$.

## 3. The Proposed Two-Dimensional Lempel–Ziv Complexity

**Figure 1:**
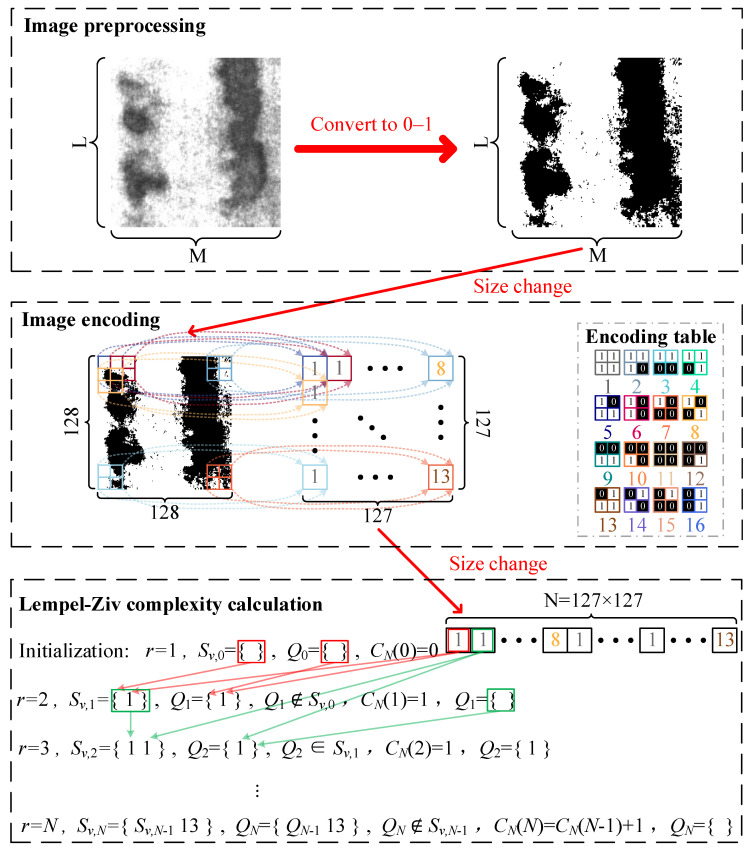
Schematic diagram of the proposed method. The calculation process of the proposed method is briefly described as follows: First, image preprocessing is performed. Then, image encoding is carried out. Finally, the LZC of the image is calculated.

Although Lempel–Ziv complexity can be effectively used for measuring the complexity of one-dimensional time series and has been used for testing anomalous patterns in one-dimensional time series, its calculation method limits its use in two-dimensional images. Therefore, the two-dimensional Lempel–Ziv complexity was proposed by combining the concept of local receptive field [48,49] in convolutional neural networks as shown in Figure 1. For an image $IM_{M\times L}$, the Lempel–Ziv complexity can be obtained as following:

Step 1: Calculate the mean of the image as follows:

(5) $$\mu_{T}=\frac{1}{M}\sum_{j=1}^{M}\frac{1}{L}\sum_{i=1}^{L}im_{ij}$$

where $im_{ij}$ is the pixel in $IM_{M\times L}$.

Step 2: Encode the image to 0–1 based on the relationship between time series and the mean $\mu_{T}$.

(6) $$is_{i}=\left\{\begin{array}{l} 1\;\;im_{ij}\geq \mu_{T}\\ 0\;\;im_{ij}<\mu_{T} \end{array}\right.$$

Step 3: Adjust the size of the image from *M* × *L* to 128 × 128 by bicubic interpolation to eliminate the influence of image size on Lempel–Ziv complexity.

Step 4: Encode the image $AI_{128\times 128}$ obtained from Step 3 based on the concept of the local receptive field to obtain the image encoding $BI_{127\times 127}$.

(7) $$bi_{kl}=\left\{\begin{array}{cc} 1 & \;\;ai_{ij}=1,ai_{ij+1}=1,ai_{i+1j}=1,a_{i+1j+1}=1\\ 2 & \;\;ai_{ij}=1,ai_{ij+1}=1,ai_{i+1j}=1,a_{i+1j+1}=0\\ 3 & \;\;ai_{ij}=1,ai_{ij+1}=1,ai_{i+1j}=0,a_{i+1j+1}=0\\ 4 & \;\;ai_{ij}=1,ai_{ij+1}=1,ai_{i+1j}=0,a_{i+1j+1}=1\\ 5 & \;\;ai_{ij}=1,ai_{ij+1}=0,ai_{i+1j}=1,a_{i+1j+1}=1\\ 6 & \;\;ai_{ij}=1,ai_{ij+1}=0,ai_{i+1j}=1,a_{i+1j+1}=0\\ 7 & \;\;ai_{ij}=1,ai_{ij+1}=0,ai_{i+1j}=0,a_{i+1j+1}=0\\ 8 & \;\;ai_{ij}=1,ai_{ij+1}=0,ai_{i+1j}=0,a_{i+1j+1}=1\\ 9 & \;\;ai_{ij}=0,ai_{ij+1}=0,ai_{i+1j}=1,a_{i+1j+1}=1\\ 10 & \;\;ai_{ij}=0,ai_{ij+1}=0,ai_{i+1j}=1,a_{i+1j+1}=0\\ 11 & \;\;ai_{ij}=0,ai_{ij+1}=0,ai_{i+1j}=0,a_{i+1j+1}=0\\ 12 & \;\;ai_{ij}=0,ai_{ij+1}=0,ai_{i+1j}=0,a_{i+1j+1}=1\\ 13 & \;\;ai_{ij}=0,ai_{ij+1}=1,ai_{i+1j}=1,a_{i+1j+1}=1\\ 14 & \;\;ai_{ij}=0,ai_{ij+1}=1,ai_{i+1j}=1,a_{i+1j+1}=0\\ 15 & \;\;ai_{ij}=0,ai_{ij+1}=1,ai_{i+1j}=0,a_{i+1j+1}=0\\ 16 & \;\;ai_{ij}=0,ai_{ij+1}=1,ai_{i+1j}=0,a_{i+1j+1}=1 \end{array}\right.$$

where i=1,2,…,127, j=1,2,…,127.

Step 5: Convert image encoding $BI_{127\times 127}$ to row vector $S_{N}=\left\{s_{1}s_{2}\ldots s_{N}\right\}$, where N=127×127.

Step 6: Initialize parameters $S_{v,0}=\left\{\;\right\}$, $Q_{0}=\left\{\;\right\}$, $C_{N}=0$, r=1.

Step 7: Take $Q_{r}=\left(Q_{r-1}s_{r}\right)$ and judge if $Q_{r}$ belongs to $S_{v,r-1}=\left\{S_{v,r-2}s_{r-1}\right\}$. If $Q_{r}\notin S_{v,r-1}$, then set $Q_{r}=\left\{\;\right\}$, $C_{N}\left(r\right)=C_{N}\left(r-1\right)+1$, r=r+1. If $Q_{r}\in S_{v,r-1}$, then set $Q_{r}=\left(Q_{r-1}s_{r}\right)$, $C_{N}\left(r\right)=C_{N}\left(r-1\right)$, r=r+1.

Step 8: Repeat Step 7 until the time series is completely covered and $C_{N}\left(N\right)$ represents the number of independent patterns in the time series.

Step 9: Normalize $C_{N}\left(N\right)$ by the following equation.

(8) $$0\leq C_{nN}=\frac{C_{N}\left(N\right)}{C_{UL}}\leq 1$$

where

(9) $$C_{UL}=\underset{N\to \infty }{\lim}C_{N}\left(N\right)=\underset{N\to \infty }{\lim}\frac{N}{\left(1-\beta \right)\log_{16}N}\approx \frac{N}{\log_{16}N}$$

## 4. Results and Discussion

This section will validate the effectiveness of the proposed method. First, one-dimensional and two-dimensional simulation signals are used to demonstrate the ability of the proposed method to reflect component changes in the signals. Then, the morphological dilation operator and Sobel operator are combined with the proposed method for defect detection, and verified with two practical cases. Finally, the limitations of the proposed method are further discussed.

### 4.1. Case Study 1: The Simulation Signal

**Figure 2:**
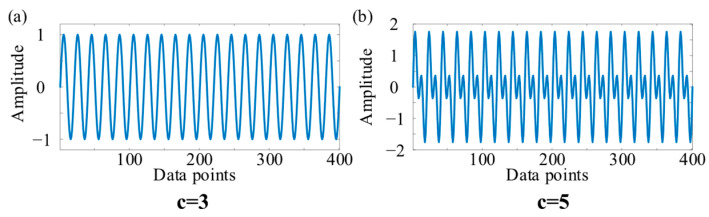
The one-dimensional simulation signal. (**a**) One frequency component in the signal. (**b**) Two frequency components in the signal.

As shown in Ref. [13], two simple one-dimensional simulation signals were used to demonstrate the ability of Lempel–Ziv complexity to reflect independent components in the signal. One simulation signal had one frequency component, while the other simulation signal had two frequency components. The waveform of the simulation signal is shown in Figure 2. And the number of independent patterns in the signal calculated using Lempel–Ziv complexity is represented by c.

It can be seen from Figure 2 that as the frequency components in the signal increased, the number of independent components calculated by Lempel–Ziv complexity also increased. For one-dimensional signals, Lempel–Ziv complexity can reflect the variation in independent patterns in the signal.

**Figure 3:**
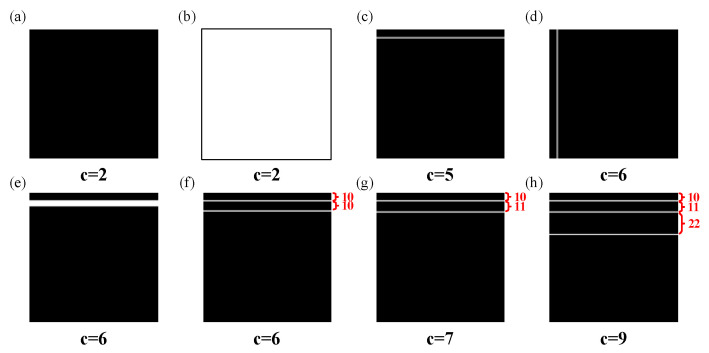
The two-dimensional simulation signal. (**a**) I1. (**b**) I2. (**c**) I3. (**d**) I4. (**e**) I5. (**f**) I6. (**g**) I7. (**h**) I8.

To demonstrate that the proposed two-dimensional Lempel–Ziv complexity can also reflect the independent patterns change in the two-dimensional signal, the following two-dimensional signal was constructed as shown in Table 1, and the number of independent patterns was calculated using the proposed two-dimensional Lempel–Ziv complexity. An image of the constructed two-dimensional signal is shown in Figure 3.

As shown in Figure 3a,b, the number of independent patterns calculated by two-dimensional Lempel–Ziv complexity in the two-dimensional signal was minimal when there were no graphics in the two-dimensional signal. In addition, the number of independent patterns calculated by two-dimensional Lempel–Ziv complexity also varied when the horizontal and vertical thickness and number of lines changed. However, when periodic components continuously appeared in the signal, the number of independent patterns calculated by two-dimensional Lempel–Ziv complexity remained unchanged. Thus, by comparing Figure 2 and Figure 3, it can be seen that the proposed two-dimensional Lempel–Ziv complexity maintained similar properties to traditional Lempel–Ziv complexity, both of which can reflect changes in the components of the signal.

Therefore, the proposed two-dimensional Lempel–Ziv complexity can be applied to defect detection problems according to the proposed two-dimensional Lempel–Ziv complexity’s ability to reflect the changes in the components of the signal. In order to eliminate the influence of background texture on the proposed two-dimensional Lempel–Ziv complexity, the dilation operator and Sobel operator were first used to preprocess the two-dimensional signal. Below, two practical cases are used to demonstrate the effectiveness of the proposed two-dimensional Lempel–Ziv complexity in defect detection.

### 4.2. Case Study 2: Type-I RSDDs Dataset

**Figure 4:**
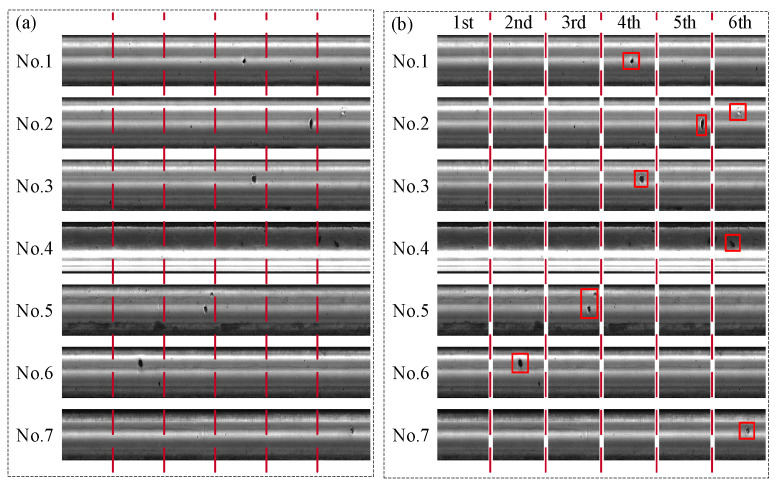
The images of Type-I RSDDs datasets. (**a**) Original images. (**b**) Divided images. The red box indicates the location of the defect in the image.

**Figure 5:**
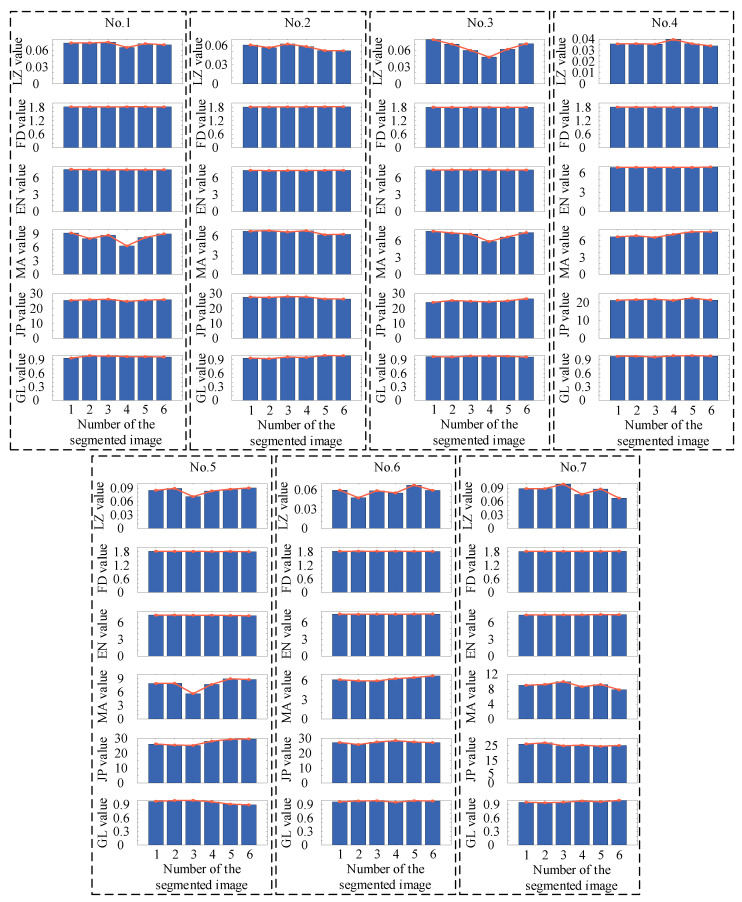
Calculation results under different methods. For the calculation results of LZ, FD, EN, MA, JP, and GL, the minimum value indicates the location of the defect.

This case was from the Type-I RSDDs dataset [50], which was captured from express rails. The size of the image in the dataset was 1000 × 160 pixels, 1260 × 160 pixels, and 1282 × 160 pixels. Then, seven sets of defective images in the dataset were selected as the samples to validate the proposed two-dimensional Lempel–Ziv complexity. As shown in Figure 4a, seven sets of images were first rotated by 90 degrees, and then 160 × 960 pixels of the seven sets of images were selected. And the above images were divided into six segments of 160 × 160 pixels images, as shown in Figure 4b. Then, the Lempel–Ziv complexity (LZ value) of the six segment images was calculated by the proposed two-dimensional Lempel–Ziv complexity. To further illustrate the advantages of the proposed two-dimensional Lempel–Ziv complexity in calculating image complexity, fractal dimension (FD value) [51], entropy (EN value) [52], machado2015 (MA value) [53], Jpg-ratio (JP value) [54], and GLCM (GL value) [55] were used for comparison. The results are shown in Figure 5.

As shown in Figure 5, for the six segmented images in each set of data, there was no significant trend change in the FD value and EN value, so defect detection cannot be performed based on the FD value and EN value. In addition, for image No. 1, the minimum position of LZ value, MA value, and JP value appeared in the fourth segment, corresponding to the segment where the defect appeared. The minimum position of GL value appeared in the first segment and the maximum position of GL value appeared in the second segment. For image No. 2, the minimum position of the LZ value, MA value, and JP value appeared in the fifth and sixth segments, corresponding to the segment where the defect appeared. The minimum position of GL value appeared in the second segment and the maximum position of the GL value appeared in the fifth and sixth segments. For image No. 3, the minimum position of LZ value and MA value appeared in the fourth segment, corresponding to the segment where the defect appeared. The minimum position of JP value appeared in the first segment, and that of the GL value appeared in the second segment and the maximum position of the GL value appeared in the third and fourth segments. For image No. 4, the minimum position of the LZ value appeared in the sixth segment, corresponding to the segment where the defect appeared. The minimum position of the MA value and GL value appeared in the third segment, and that of the JP value appeared in the fourth segment. The maximum position of GL value appeared in the fifth segment. For image No. 5, the minimum position of LZ value, MA value, and JP value appeared in the third segment, corresponding to the segment where the defect appeared. The minimum position of the GL value appeared in the sixth segment and the maximum position of GL value appeared in the third segment. For image No. 6, the minimum position of the LZ value and JP value appeared in the second segment, corresponding to the segment where the defect appeared. The minimum position of the MA value appeared in the third segment, and that of the GL value appeared in the fourth segment. The maximum position of GL value appeared in the second and fifth segments. For image No. 7, the minimum position of the LZ value and the MA value appeared in the sixth segment, corresponding to the segment where the defect appeared. The minimum position of the JP value appeared in the fifth segment, and that of the GL value appeared in the second segment. The maximum position of the GL value appeared in the sixth segment. Thus, from the above results, it can be seen that although the position of the defects can be determined by the minimum MA value, minimum JP value, and maximum GL value for some samples, only the minimum LZ value corresponds to the segmentation of defects for all samples.

### 4.3. Case Study 3: AITEX Dataset

**Figure 6:**
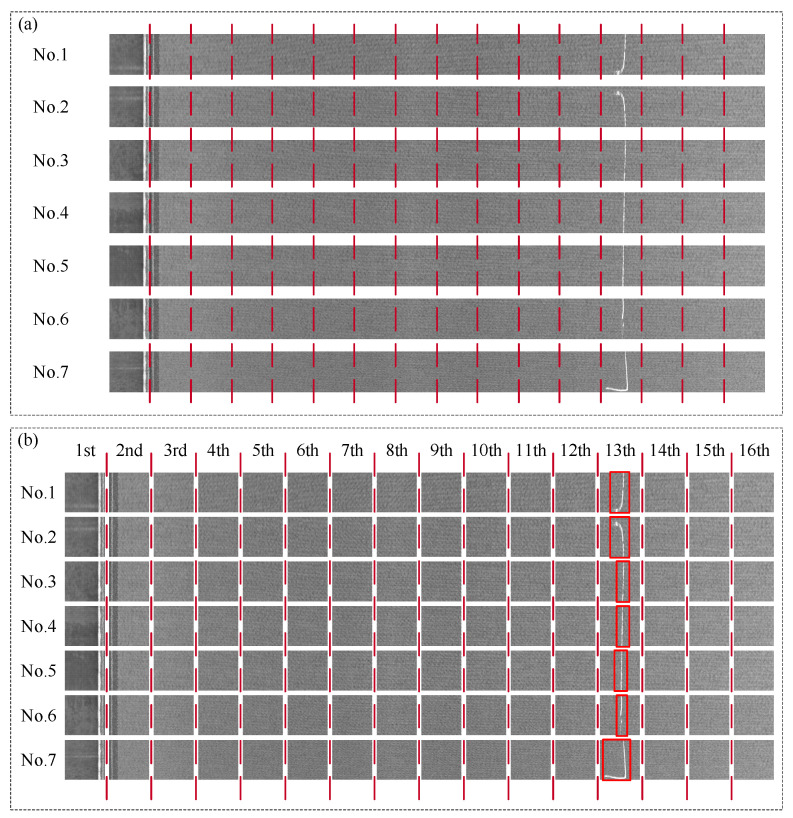
The images of AITEX datasets. (**a**) Original images. (**b**) Divided images. The red box indicates the location of the defect in the image.

**Figure 7:**
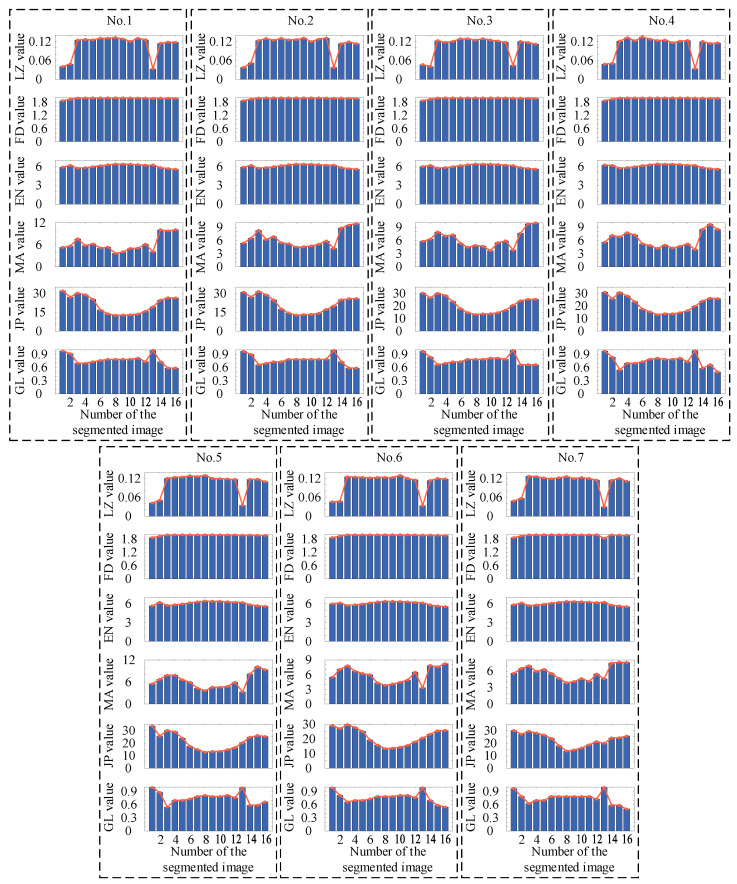
Calculation results achieved using different methods. For the calculation results of LZ, FD, EN, MA, JP, and GL, the minimum value indicates the location of the defect.

This case was from the AITEX dataset [56], which was the dataset of fabric defects. The size of the image in the dataset was 256 × 4096 pixels. Then, seven sets of defective images in the dataset were selected as the samples to validate the proposed two-dimensional Lempel–Ziv complexity. We divided the above images into 16 segments of 256 × 256 pixels images as shown in Figure 6a,b. Then the LZ value, FD value, EN value, MA value, JP value, and GL value were calculated as shown in Figure 7.

As shown in Figure 7, for the LZ value, the minimum position of all the samples appeared in the 13th segment, corresponding to the segment where the defect appeared. For FD value, only for image No. 7, the minimum position appeared in the 13th segment, corresponding to the defect segment, while the minimum position of the remaining samples appeared in the first segment. For EN value, the minimum position appeared in the 16th segment, and the maximum position appeared in the eighth segment, none of which corresponded to the defect segment. For the MA value, the minimum position of image No. 2, No. 4, No. 5, and No. 6 appeared in the 13th segment, corresponding to the defect segment; the minimum position of image No. 1 and No. 7 appeared in the eighth segment, and the minimum position of image No. 3 appeared in the 10th segment. For the JP value, the minimum position of all samples appeared in the eighth segment. The maximum positions of image No. 1, No. 3, No. 4, No. 5, and No. 7 appeared in the first segment and that of the remaining images appeared in the second segment. For the GL value, the maximum position of all the samples appeared in the 13th segment, corresponding to the segment where the defect appeared. Therefore, from the above results, it can be seen that the position of defects can be determined based on the minimum LZ value and the maximum GL value.

### 4.4. Discussion

**Figure 8:**
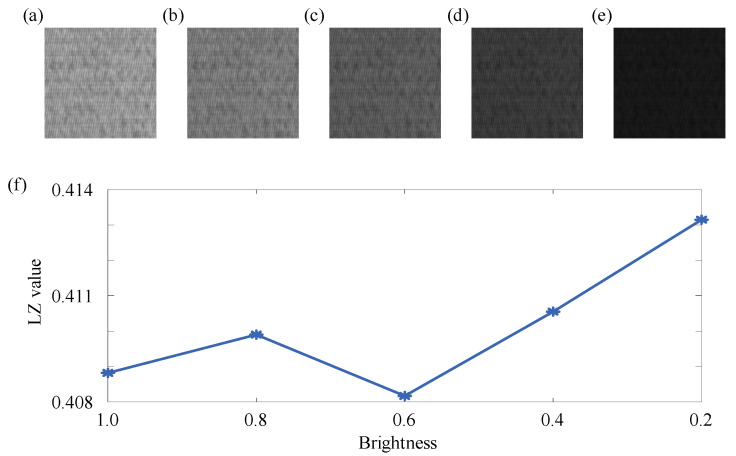
The image under different brightness and the result of two-dimensional Lempel–Ziv complexity: (**a**) 1; (**b**) 0.8; (**c**) 0.6; (**d**) 0.4; and (**e**) 0.2. (**f**) The result of two-dimensional Lempel–Ziv complexity.

**Figure 9:**
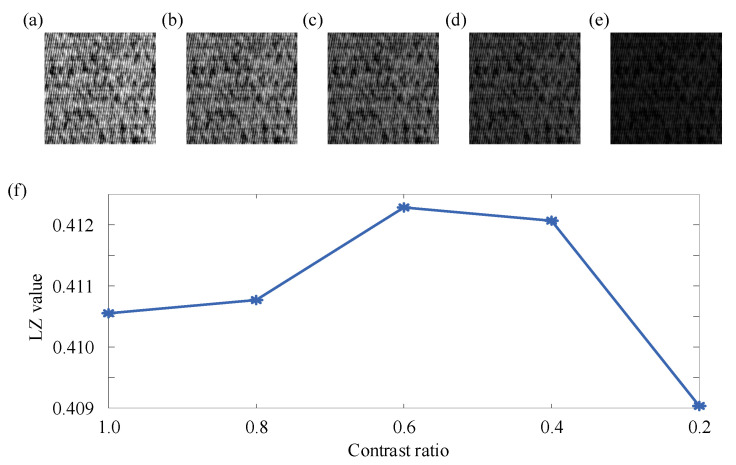
The image under different contrast ratios and the result of two-dimensional Lempel–Ziv complexity: (**a**) 1; (**b**) 0.8; (**c**) 0.6; (**d**) 0.4; (**e**) 0.2. (**f**) The result of two-dimensional Lempel–Ziv complexity.

**Figure 10:**
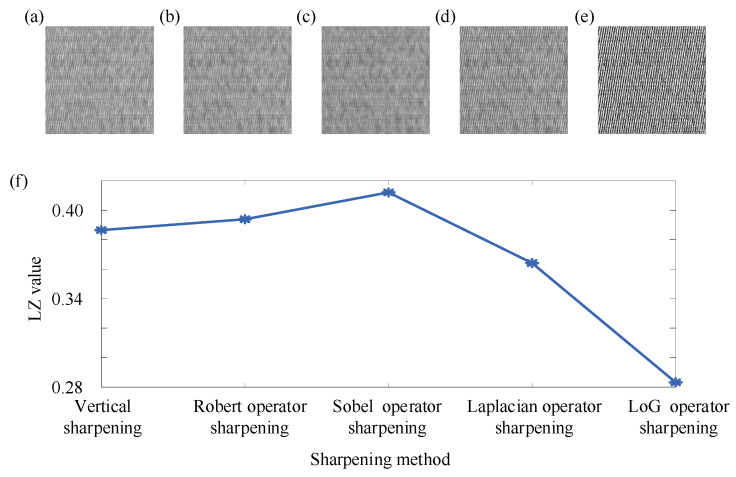
The image under different sharpening method and the result of two-dimensional Lempel–Ziv complexity. (**a**) Vertical sharpening. (**b**) Robert operator sharpening. (**c**) Sobel operator sharpening. (**d**) Laplacian operator sharpening. (**e**) LoG operator sharpening. (**f**) The result of two-dimensional Lempel–Ziv complexity.

Through the above simulation signals and two practical cases, it can be seen that the proposed two-dimensional Lempel–Ziv complexity can effectively reflect the changes in non-periodic components in the image, and combined with the dilation operator and Sobel operator, it can be effectively used for defect detection. However, due to the sensitivity of the proposed two-dimensional Lempel–Ziv complexity to non-periodic components in the image, there were certain limitations when used alone. The brightness, contrast, and texture edge sharpening of the image would affect the two-dimensional Lempel–Ziv complexity. Therefore, taking the 16th segment image of No. 1 in case study 2 as an example, the effects of brightness, contrast, and texture edge sharpening on the two-dimensional Lempel–Ziv complexity are illustrated below. Firstly, the above example image was multiplied by different coefficients (1, 0.8, 0.6, 0.4, 0.2) to simulate different levels of brightness, as shown in Figure 8a–e. The pixel values of the above example image were adjusted to the range of 0 to α (α = 1, 0.8, 0.6, 0.4, 0.2) to simulate different contrast ratios, as shown in Figure 9a–e. The above example image was processed by different sharpening methods (vertical sharpening [57], Robert operator sharpening [58], Sobel operator sharpening [59], Laplacian operator sharpening [60], and LoG operator sharpening [61]) to simulate different edge sharpening, as shown in Figure 10a–e. Then, the two-dimensional Lempel–Ziv complexity of the above images was calculated separately, as shown in Figure 8f, Figure 9f and Figure 10f.

As shown in Figure 8f, there were certain differences in LZ values under different brightnesses. The reason for this phenomenon was that the texture in the above images was relatively complex, and the changes between all pixels were relatively continuous. Therefore, during the normalization process of images with different brightnesses, there was a situation which the values near the mean of all pixels fluctuated up and down around the mean of all pixels due to rounding errors, which further affected the encoding of the image and the LZ value. Therefore, for complex texture images with small differences in pixel values, changes in brightness will affect the LZ value.

As shown in Figure 9f, there were certain difference in LZ values under different contrast ratios. The reason for this phenomenon was basically consistent with the different LZ value under different brightnesses mentioned above. This was mainly because there were pixels with small differences in complex textures. Therefore, during the process of changing the range of pixel values, rounding errors caused the values near to the mean of all pixels to fluctuate up and down around the mean of all pixels, which further affected the encoding and LZ values of the image. Therefore, for complex texture images with small differences in pixel values, changes in contrast ratio will affect the LZ value.

As shown in Figure 10f, there were certain difference in LZ values under different sharpening method. As shown in Figure 10a–e, the images processed by different sharpening methods were completely different, except for the overall texture style. Because sharpening caused the changes in the content of the image, there was a difference in the LZ value. Therefore, when the content of the image changed, it also had an impact on the LZ value.

**Figure 11:**
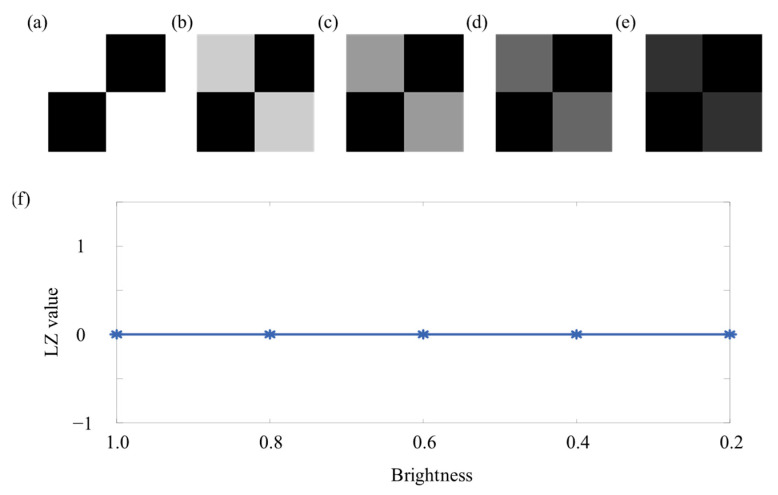
The image under different brightness and the result of two-dimensional Lempel–Ziv complexity: (**a**) 1; (**b**) 0.8; (**c**) 0.6; (**d**) 0.4; (**e**) 0.2. (**f**) The result of two-dimensional Lempel–Ziv complexity.

From the above discussion, it can be seen that for complex textures with small differences between pixels, changes in brightness and contrast will have an impact on the LZ value. To make the discussion more comprehensive, below, we present a simple texture with significant differences between pixels to illustrate the situation under different brightnesses and contrast ratios. We constructed an image of 128 × 128 pixels as shown in Figure 11a, where rows 1 to 64 and the columns 1 to 64 and rows 65 to 128 and columns 65 to 128 are represented by a value of 1, and the remaining positions are represented by 0. Because for the texture in Figure 11, the changes in contrast ratio and brightness using the above method are the same, only the LZ values of the constructed simple texture at different brightness were calculated as shown in Figure 11f.

As shown in Figure 11f, for simple textures with significant differences between pixels, the LZ value remained unchanged at different brightness. This was because there were only two types of texture in the image, light and dark, and there was a significant difference in pixel values between the two textures. Even if the range of light and dark values was changed, this difference would not change. Therefore, the normalization results of the image will not change, and the LZ value will not change.

From the above discussion, it can be seen that the proposed two-dimensional Lempel–Ziv complexity is sensitive to small changes in the content of the image. For the simple textures with significant differences between pixels, the proposed two-dimensional Lempel–Ziv complexity can effectively reflect the changes in texture in the image. However, for complex textures with small differences between pixels, the proposed two-dimensional Lempel–Ziv complexity would be affected by the background texture. Therefore, it is necessary to preprocess the image by using the dilation operator to enhance the texture and the Sobel operator to extract texture edges to eliminate the influence of brightness and contrast on the LZ value.

## 5. Conclusions

This paper proposed a two-dimensional Lempel–Ziv complexity by combining the concept of local receptive field in convolutional neural networks. This extends the application scenario of LZC from one-dimensional time series to two-dimensional images, further broadening the scope of application of LZC. The pixels and size of the image were first normalized to eliminate the impact of image size on Lempel–Ziv complexity. Then, the image was encoded according to the sorting of normalized values within the 4 × 4 region. Next, the encoding result of the image was rearranged into a vector by row. Finally, the Lempel–Ziv complexity of image can be obtained based on the rearranged vector. The proposed method was further used for defect detection in conjunction with the dilation operator and Sobel operator. Through the verification of the one-dimensional and two-dimensional simulation signals and two practical cases, it was proved that the proposed method was sensitive to small changes in the content of the image and can be used for defect detection.

However, the proposed method still has the following limitations. For simple textures with significant differences between pixels, the proposed two-dimensional Lempel–Ziv complexity can effectively reflect the changes in texture in the image. However, for complex textures with small differences between pixels, the proposed two-dimensional Lempel–Ziv complexity would be affected by the background texture. Therefore, the application of the proposed method to the defect detection of complex textures deserves further research.

## Figures and Tables

**Table 1:** The constructed two-dimensional signal information.

Serial Number of 2D Signal	Description
I1	zeros (128, 128)
I2	ones (128, 128)
I3	zeros (128, 128) and the 10th row is 1
I4	zeros (128, 128) and the 10th column is 1
I5	zeros (128, 128) and the 10th, 11th, 12th, 13th, 14th, and 15th rows are 1
I6	zeros (128, 128) and the 10th and 20th row are 1
I7	zeros (128, 128) and the 10th and 21st rows are 1
I8	zeros (128, 128) and the 10th, 21st, and 43rd rows are 1

## Data Availability

The data in the manuscript is a publicly available dataset. “Type-I RSDDs dataset” at https://gitcode.com/open-source-toolkit/1c98b (accessed on 1 December 2023). “AITEX dataset” at https://www.aitex.es/afid/ (accessed on 1 December 2023).
